# Intercomparisons of Nine Sky Brightness Detectors

**DOI:** 10.3390/s111009603

**Published:** 2011-10-11

**Authors:** Peter den Outer, Dorien Lolkema, Marty Haaima, Rene van der Hoff, Henk Spoelstra, Wim Schmidt

**Affiliations:** 1 National Institute for Public Health and the Environment, A. van Leeuwenhoeklaan 9, 3720 BA Bilthoven, The Netherlands; E-Mails: dorien.lolkema@rivm.nl (D.L.); marty.haaima@rivm.nl (M.H.); rene.van.der.hoff@rivm.nl (R.H.); 2 Lumineux Consult, Landgraafstraat 96, 6845 ED Arnhem, The Netherlands; E-Mail: henk@lumineux-consult.com; 3 Sotto le Stelle, Merwedeplantsoen 27, 3522 JS Utrecht, The Netherlands; E-Mail: wim.schmidt@sotto.nl

**Keywords:** artificial lighting, intercomparison, intercalibration, light pollution, night sky brightness, Sky Quality Meter

## Abstract

Nine Sky Quality Meters (SQMs) have been intercompared during a night time measurement campaign held in the Netherlands in April 2011. Since then the nine SQMs have been distributed across the Netherlands and form the Dutch network for monitoring night sky brightness. The goal of the intercomparison was to infer mutual calibration factors and obtain insight into the variability of the SQMs under different meteorological situations. An ensemble average is built from the individual measurements and used as a reference to infer the mutual calibration factors. Data required additional synchronization prior to the calibration determination, because the effect of moving clouds combined with small misalignments emerges as time jitter in the measurements. Initial scatter of the individual instruments lies between ±14%. Individual night time sums range from −16% to +20%. Intercalibration reduces this to 0.5%, and −7% to +9%, respectively. During the campaign the smallest luminance measured was 0.657 ± 0.003 mcd/m^2^ on 12 April, and the largest value was 5.94 ± 0.03 mcd/m^2^ on 2 April. During both occurrences interfering circumstances like snow cover or moonlight were absent.

## 1. Introduction

Today, the World at night is bright. Pictures from space show us beautifully and strikingly the modern human footprint by how we illuminate our night. One of the brightest spots on these maps comes from The Netherlands, a very densely populated area. Although the need for artificial light in populated areas is beyond question, there are also adverse effects of night time light on flora and fauna as well as on humans. For example, it affects the foraging, reproductive and migration behaviour of a number of nocturnal animals such as insects, bats, amphibians and birds. Furthermore, it changes prey-predator relationships, affects animal natural rhythms, disrupts physiological processes in plants [1–3] and adverse effects on the human metabolism have also been found [4].

Because in the Netherlands industry, nature reserves, residential areas and greenhouses lie closely together, both the positive and negative aspects of artificial light at night are strongly intermingled. Currently, actual levels and the variability of local night sky brightness are not well known. Therefore, we have set up a monitoring network of light meters distributed across the Netherlands. The network should give insight into the actual values and the variability in time and place of night sky brightness. The measurements can also be utilised for satellite (DMSP-OLS [5–7] and VIIRS [8]) data validation purposes and model-output [5–7,9,10]. We use Sky Quality Meters (SQMs, type SQM-Lens Ethernet, Unihedron, Grimsby. ON, Canada) as they are a low-cost light meter and are widely used by astronomers to measure the “quality” of the night sky. For monitoring night time luminances from the perspective of light abundance, they are already used in several countries worldwide (Italy, Spain, Mexico, Canada, and Namibia) [11]. In Germany, SQM measurements are performed in Berlin [12] and preparations are under way to build an SQM-based network [13]. Additionally, SQMs are used by Lumineux Consult and Sotto le Stelle, both operating in the Netherlands.

In this paper, we focus on an intercomparison campaign that was held before the SQMs were distributed to their intended monitoring locations. The six instruments of RIVM where joined by one SQM operated by Sotto le Stelle and two operated by Lumineux Consult. The goal of the intercomparison was to infer mutual calibration factors and obtain insight into the variability of the SQMs under different meteorological situations. The intercomparison campaign was held in spring 2010 at the Cabauw Experimental Site for Atmospheric Research (CESAR) [14]. Today’s monitoring nine locations, including those of Sotto le Stelle and Lumineux Consult, are in four nature reserve areas, with an expected low average luminance, three in cities, one in a greenhouse area, and one at CESAR, which is a rural area.

## 2. Measurements

### 2.1. Sky Quality Meter

An SQM measures the sky quality in magnitudes per square arc second, ranging from 24 mag/arc s^2^ (almost complete darkness) to 0 mag/arc s^2^ (occurring during dawn and dusk). The aperture of the instrument is approximately 20°. We measured the angular response of one instrument. A Gaussian fit delivered an FWHM of 17.2° and 18.0°, for two perpendicular directions. The incoming light falls through an acrylic flat window (the SQMs of Lumineux Consult have domes) and through an infrared filter (HOYA CM-500) on a solid state light-to-frequency converter (TSL237). The spectral response encompasses the photopic eye response [15] but is more sensitive towards the blue wavelength [16]. The SQMs have been calibrated by the manufacturer and the readings are internally temperature corrected. The output of the SQM is ultimately expressed in Luminances using the conversion supplied by the manufacturer:

(1) $$\text{Luminance}\;[\text{cd}/m^{2}]=10.8\times 10^{4}\times 10^{\left(-0.4*\text{magnitude}\right)}$$

Since the spectral sensitivity of an SQM does not match the photopic eye response, care should be taken when applying the derived luminances as such. However, within the focus of this paper it does not add any uncertainties since we intercompare SQMs of the same type.

During the measurement campaign, we placed the SQMs at the same height and within 2 m distance, the central position of the light meters was 51.9682°N, 4.9293°E. All SQMs pointed at zenith; a residual inaccuracy of about 5 degrees can, however, not be eliminated. The instruments were fully operational from 1 April 2011 until 7 May 2011, and 24 hours a day. Eight SQMs were synchronised by one computer, and one was read-out by a separate one. The clocks on both computers were synchronized through the CESAR network. The 10-second readings were stored. One-minute integrated values were constructed from which the mutual calibration is derived. We use the convention that one night time measurement starts at noon and lasts until 11:59 am the next day, each night time measurement will be indicated by the date of the day containing the evening e.g., the measurement during the night from 1 April to 2 April, is indicated by measurement 1 April. The Coordinated Universal Time (UTC) is used. For night time plots we apply UTC + 24 for the am hours to obtain a continuous time scale.

### 2.2. CESAR

The intercomparison was held at the Cabauw Experimental Site for Atmospheric Research. This site is owned and maintained by the Royal Netherlands Meteorological Institute, KNMI, and home to the CESAR-consortium that performs advanced atmospheric research [14]. A high number of atmospheric parameters are monitored at CESAR through passive and active observations. CESAR is located in the western part of the Netherlands. The surrounding landscape is open and consists mainly of pasture and small villages. Two very small villages, Cabauw (pop. 700) and Lopik (pop. 5,400), are located nearby at about 2 km. The village of Schoonhoven (pop. 12,000) is located at 5 km distance, Gouda (pop. 71,000) and Nieuwegein (pop. 61,000) are located at a distance of about 12 km from the measurement site. Larger cities are located at a distance of 20 km (Utrecht) and 30 km (Rotterdam). The closest illuminated motorway is located at a distance of 6 km. During night time, the influence of sunlight could not be detected for Solar Elevation Angles (SEA) smaller than −15° [16]. For the analysis presented here, therefore, we exclusively use data points for which the solar elevation angle is smaller than −15°.

## 3. Analysis Techniques

### 3.1. Data Analysis

**Figure 1.**
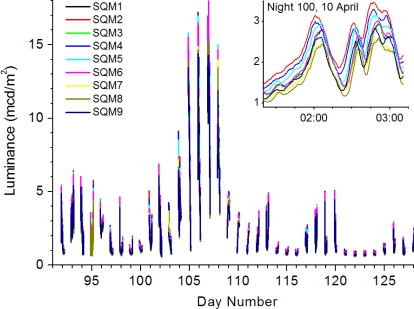
Night time luminances as measured by the nine SQMs during the intercomparison campaign at Cabauw, 1 April until 8 May, the Netherlands. Inset shows a detail of measurements, time is indicated in hours.

Figure 1 shows the measured time series for the whole intercomparison campaign. A high correlation between the instrument readouts is readily seen. We can easily identify the period with the moon above the horizon, Moon Elevation Angle (MEA) > 0°, and for nights with MEA < 0°, periods with small luminances and moderate luminances due to the presence of clouds. Zooming in, a time jitter in the signals is observed. Although an electronic origin for the observed time jitter cannot be excluded at this moment, it is most likely caused by small directional misalignments of the detectors, *i.e.*, the field of view is not centred at zenith for all detectors. Consequently, isolated clouds, giving rise to an increased luminance, will be detected at slightly different times. Of course, the observed time jitter should then depend on wind speed combined with cloud base height and wind direction, but such a detailed analysis is beyond the scope of this paper. In either case, the jitter should be eliminated before the mutual calibration factors can be inferred. Additionally, we disregard any mismatches in wavelength response as a possible source of the observed time jitter, since we compare nine SQMs, and not nine different detection devices. We have arbitrarily set the measurements of SQM6 as the time standard and determined the time dependent time shifts for the remaining eight instruments relative to this instrument. We first interpolated the stored 10-s readings to 1-s readings. Next, for steps of 30 min, we determined the time shift by maximizing the correlation between each instrument and the chosen reference instrument within time windows of 60 min centred on the time step. The absolute time shifts found varied from 0 to 180 s, which eventually rules out an electronic origin as instruments are readout by the same computer. The synchronized data were again integrated to 1-minute average values and used in the rest of the analysis to obtain the mutual calibration factors.

We have adopted a procedure that has been used in intercomparison campaigns in the field of spectrally resolved ultraviolet radiation measurements. By ensemble averaging a reference data set is inferred to which the individual instruments can be compared [17,18]. Recently the same procedure was used in an intercomparison of ultraviolet radiation reconstruction models [19]. Such an elaborate procedure is required, since we do not have access to a “true” luminance standard for the intercomparison period and we cannot *a priori* deem one instrument better than others.

The key parameter for each instrument will be the averaged value of its assigned so-called median multiplication factors (MMF). An MMF is derived for each night and for each instrument separately and independently. It is the required magnitude of the multiplication factor to the measurements of one instrument so that the median of this subset coincides with the median of all measurements for that night. An instrument with too low a calibration will consequently produce an array of high MMFs and too high a calibration will result in an array with small MMF values. The stability of an instrument is reflected in the variability of its MMFs. The sought reference is now the weighted average of scaled measurements, where the scaling factor for each instrument is delivered by its averaged MMFs and its statistical weight is taken to be inversely proportional to the standard deviation in the average squared. The procedure provides also in filtering the out layers.

## 4. Results and Discussion

**Figure 2.**
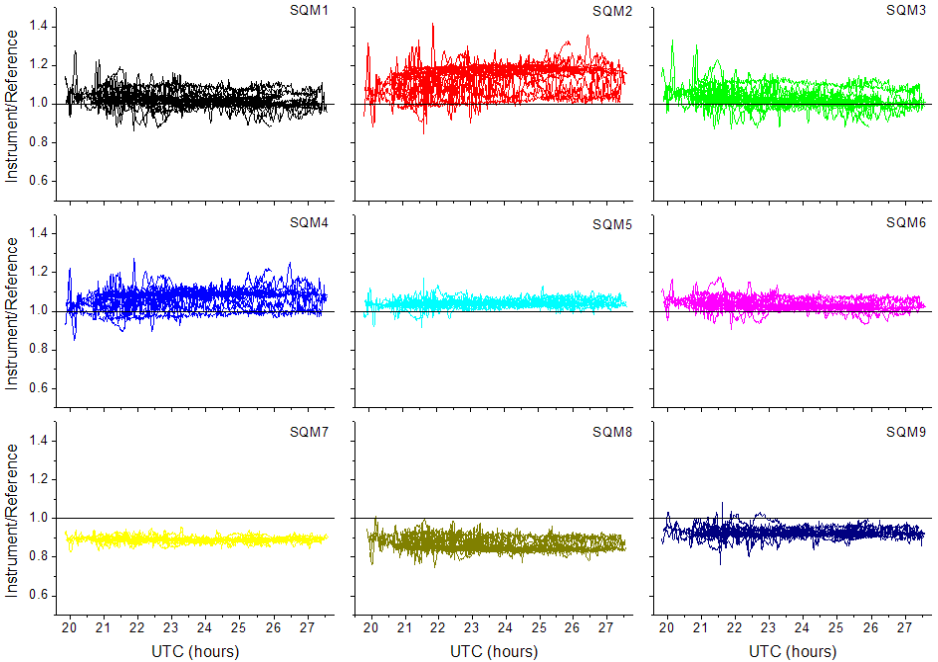
Measured luminances divided by the reference as a function of time. For all panels holds: SEA < −15° and MEA < 0°, UTC + 24 is used for the morning hours.

In Figure 2 we plot the measured luminance divided by the constructed reference as a function of time (hours) for the whole intercomparison period. Clearly, different behaviour of the SQMs is found. We can roughly rank the SQM as follows: 5, 6, 7, 9 are stable, 1, 3, 8 show some multilevel behaviour, and 2, 4 show the most scatter. This is further underpinned by the found standard deviations given in Table 1. In this table the averaged ratios of measured to reference are given. Nights with moonlight give rise to the large standard deviations in the means and, not unexpected, variations during clear nights are the smallest. The instruments have a scatter of less than ±14%, for MEA < 0°.

Deviations of night time sums, the integrated luminance during the set limit of the SEA, range from −16% to +20%. Plotting the same data but now as direct correlation plots, reference *vs.* instruments, allows for a derivation of intercalibration factors for each instrument by fitting straight lines. Zero offset could not be a constraint in the fitting procedure, and hence each instrument was assigned an offset and a scaling factor, see Table 2.

**Figure 3.**
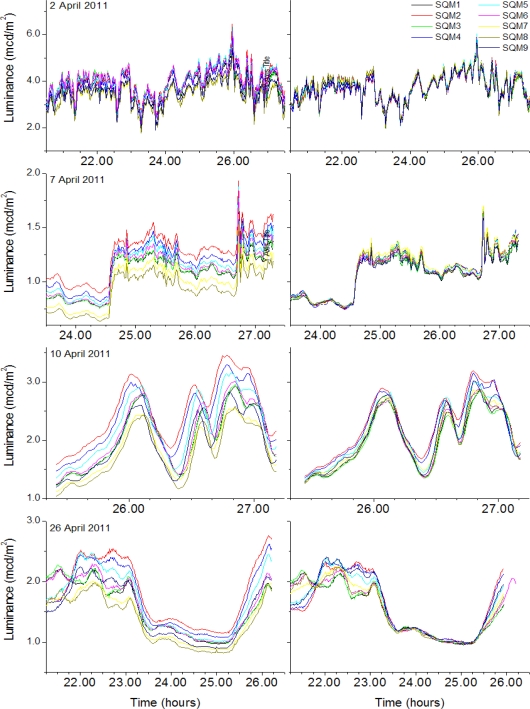
Uncorrected (left column) and intercalibrated (right column) luminances as a function of time for some exemplary nights. The same scale applies to rows only, UTC + 24 is used for the morning hours.

By applying these intercalibration factors, the different data sets almost merge to one curve, as shown in Figure 3. This figure shows both situations for a number of exemplary nights, before and after intercalibration. It could be argued that a simple scaling would have been be sufficient, as the top two panels seem to indicate, but on closer examination, the order of the instruments is interchanged, which would have introduced ambiguity in the sought intercalibration factors. Moreover, time shifts had to be eliminated, clearly visible in the third row. To overcome these obstacles, the more elaborated procedure was followed. The corresponding values of Table 1 are given in Table 3. After the intercalibration the initial scatter of ±14% is reduced to 0.5%, and deviations of night time sums range now from −7% to +9%, which was −16% to 20%. Due to moonlight the “All Nights” column, and even more pronounced in the ‘Moon lit nights only’ column, show large variations in detected signals. This is caused by the fact that the moon is quite bright and moonlight may be detected through side cloud reflections as well. The latter effect has an inherent high spatial and temporal variability. It would require extremely accurate matching field of views of the nine SQMs to obtain similar results in this column compared to the first two columns. Alternatively, we can reverse the argument and state that a good agreement of the measurements under moon lit nights ensures a high accuracy of the alignment of the detectors.

The smallest luminance was measured at 12 April: 0.657 ± 0.003 mcd/m^2^, the highest value on 2 April: 5.94 ± 0.03 mcd/m^2^. Including moon lit nights, the highest values measured was 16.6 ± 0.1 mcd/m^2^. Averages per night are within the boundaries from 0.75 to 3.7 mcd/m^2^, for SEA < −15° and MEA < 0°.

Focusing on the first six SQMs, purchased together by RIVM, a noticeable grouping occurs in the performance: 6 and 5 score best, then 1 and 3, and finally 2 and 4. This grouping appears to correlate with the manufacturer batch numbers of the electronic integrated circuit that turn out to be paired in the same manner.

## 5. Conclusions

Nine Sky Quality Meters were intercompared during a one-month period and mutual calibration factors have been derived. The raw data of the individual instruments agreed within 14%, with deviations of individual nights sums ranging from −16% to +20%. After intercalibration had been applied this is reduced to 0.5%, with deviations ranging from −7% to +9%. A straightforward derivation of the intercalibration factors was hampered by a time jitter and a ‘non-linear behaviour’ of the instruments, e.g., the ranking of the instrument with respect to the measured luminances varied over the nights. The observed time jitter is an artefact caused by drifting clouds combined with small misalignments of the field of view of the detectors. However, removal of the time jitter was crucial to obtain the found agreement of the SQMs.

During the intercomparison, the highest measured luminance was 17 mcd/m^2^ and mainly due to moonlight, excluding moonlight contributions, the highest luminances at CESAR lay around 6 mcd/m^2^. On clear, cloudless nights, this reduces to about 0.6 mcd/m^2^.

The good agreement between the nine instruments was derived after a successful scaling of the data. It should be noted that being able to perform such a scaling in the first place is a good achievement for straight forward, ready to use light meters and enables us to truly operate these type of devices in a monitoring fashion. Long-term stability of the instruments with this high accuracy is yet to be determined and will be the subject of future (technical) studies.

## Figures and Tables

**Table 1.** Average ratios of measurements to reference and standard deviations.

**Days with Instrument**	**Clear Nights (N = 2,379)**	**MEA < 0° (N = 7,952)**	**All Nights (N = 13,790)**
SQM1	1.005 ± 0.012	1.02 ± 0.04	1.01 ± 0.05
SQM2	1.168 ± 0.017	1.14 ± 0.06	1.14 ± 0.07
SQM3	1.000 ± 0.010	1.02 ± 0.04	1.01 ± 0.05
SQM4	1.079 ± 0.010	1.07 ± 0.04	1.09 ± 0.06
SQM5	1.027 ± 0.008	1.04 ± 0.02	1.05 ± 0.02
SQM6	1.014 ± 0.006	1.03 ± 0.03	1.03 ± 0.03
SQM7	0.880 ± 0.004	0.89 ± 0.01	0.91 ± 0.04
SQM8	0.835 ± 0.006	0.86 ± 0.03	0.87 ± 0.04
SQM9	0.908 ± 0.007	0.92 ± 0.02	0.92 ± 0.03

**Table 2.** Determined intercalibration factors.

**Instrument**	**Offset**	**Scaling**
SQM1	+0.0261 ± 0.0016	0.9610 ± 8.5E−4
SQM2	−0.1123 ± 0.0024	0.9604 ± 0.0012
SQM3	+0.0446 ± 0.0017	0.9447 ± 9.6E−4
SQM4	−0.0585 ± 0.0020	0.9785 ± 0.0010
SQM5	+0.0155 ± 0.0007	0.9503 ± 3.8E−4
SQM6	+0.0323 ± 0.0010	0.9453 ± 5.2E−4
SQM7	−0.0005 ± 0.0006	1.1254 ± 3.6E−4
SQM8	+0.0760 ± 0.0011	1.0927 ± 6.7E−4
SQM9	+0.0284 ± 9.4E−4	1.0580 ± 5.4E−4

**Table 3.** Average ratios of measurements to reference after intercalibration. Moon lit nights are above 6 mcd/m^2^.

**Days with**	**Clear Nights (N = 2,383)**	**MEA < 0° (N = 7,952)**	**All Night (N = 13,788)**	**Moon lit nights only (N = 1,218)**	**Extrema (mcd/m2) (all nights)**	
					**min**	**max**
SQM1	0.999 ± 0.012	1.001 ± 0.032	0.99 ± 0.05	0.95 ± 0.05	0.65	16.63
SQM2	1.004 ± 0.014	1.002 ± 0.052	1.02 ± 0.06	1.02 ± 0.07	0.69	16.45
SQM3	0.996 ± 0.012	1.001 ± 0.033	0.96 ± 0.05	0.88 ± 0.05	0.65	15.27
SQM4	1.002 ± 0.009	1.000 ± 0.043	1.01 ± 0.06	1.03 ± 0.07	0.66	17.12
SQM5	1.001 ± 0.008	1.001 ± 0.019	1.01 ± 0.02	1.03 ± 0.02	0.67	17.66
SQM6	0.998 ± 0.009	1.001 ± 0.019	0.99 ± 0.03	0.98 ± 0.04	0.65	17.17
SQM7	1.000 ± 0.003	1.000 ± 0.015	1.02 ± 0.04	1.08 ± 0.05	0.66	17.57
SQM8	0.999 ± 0.007	1.001 ± 0.021	1.01 ± 0.04	1.07 ± 0.04	0.65	17.86
SQM9	1.001 ± 0.008	1.000 ± 0.019	0.99 ± 0.03	0.97 ± 0.04	0.65	15.70
